# Chemical–Electrochemical Process Concept for Lead Recovery from Waste Cathode Ray Tube Glass

**DOI:** 10.3390/ma14061546

**Published:** 2021-03-22

**Authors:** Árpád Imre-Lucaci, Melinda Fogarasi, Florica Imre-Lucaci, Szabolcs Fogarasi

**Affiliations:** 1 Department of Chemical Engineering, Faculty of Chemistry and Chemical Engineering, Babeş-Bolyai University, 11 Arany Janos Street, 400028 Cluj-Napoca, Romania; arpad.imre@ubbcluj.ro; 2 Department of Food Engineering, University of Agricultural Sciences and Veterinary Medicine of Cluj-Napoca, Calea Mănăstur 3-5, 400372 Cluj-Napoca, Romania; 3 Interdisciplinary Research Institute on Bio-Nano-Sciences, Babeş-Bolyai University, 42 Treboniu Laurian Street, 400271 Cluj-Napoca, Romania

**Keywords:** cathode ray tube, lead recovery, lead leaching and electrodeposition, environmental assessment

## Abstract

This paper presents a novel approach for the recovery of lead from waste cathode-ray tube (CRT) glass by applying a combined chemical-electrochemical process which allows the simultaneous recovery of Pb from waste CRT glass and electrochemical regeneration of the leaching agent. The optimal operating conditions were identified based on the influence of leaching agent concentration, recirculation flow rate and current density on the main technical performance indicators. The experimental results demonstrate that the process is the most efficient at 0.6 M acetic acid concentration, flow rate of 45 mL/min and current density of 4 mA/cm^2^. The mass balance data corresponding to the recycling of 10 kg/h waste CRT glass in the identified optimal operating conditions was used for the environmental assessment of the process. The General Effect Indices (GEIs), obtained through the Biwer Heinzle method for the input and output streams of the process, indicate that the developed recovery process not only achieve a complete recovery of lead but it is eco-friendly as well.

## 1. Introduction

In the recent years there has been a major concern to limit the risks associated with the manufacture of electrical and electronic equipment, the management of waste electrical and electronic equipment (WEEE) in order to minimize the negative impact on the environment [1,2,3]. In this regard, the European Union Regulations on Electronic Waste, WEEE Directive 2002/96/EC, EU WEEE DIRECTIVE 2012/19/EU and RoHs 2002/95/EC, regulate the responsibility of the member countries for the collection, use, recycling and recovery of electronic waste [4]. Rapid growth in production and technical development in electronics involves the accelerated replacement of outdated electronics equipment and accumulation of large amounts of harmful WEEE [5,6], including waste cathode-ray tube (CRT) glass from old televisions and computer monitors [7].

A CRT is composed of two different types of glass, from which one is used for the funnel and neck sections, characterized by high levels of lead oxide and another used for the screen which is typically a non-leaded glass that contains high levels of barium oxide [8]. Recycling of lead from waste CRT glass is an important issue because lead is classified as a neurotoxin that can accumulate in the soft tissues and bones, causing serious health issues [9,10,11]. The high content of lead oxide (23%) in CRT funnel glasses is an important factor that limits its landfill storage and the recycling process, as result many states have passed bans on putting CRTs in landfills or incinerators [12,13]. Additionally, CRT funnel glass, due to its composition, is unsuitable for applications where metal oxides could leach into food products or ground water [14].

Conventional CRT glass recycling is carried out in a closed-loop, where waste glass, after an appropriate removal of metal and luminophore contaminants, is utilized during manufacturing of new CRTs [15,16]. However, the above-mentioned recycling method is insufficient, as technology develops and modern liquid crystal display (LCD), plasma or light-emitting diode (LED) screens are introduced, the demand for CRT glass decreases [17]. As a result, it is necessary to provide the industry with new technical solutions for the processing of waste glass which led to other products than CRTs [18]. The most common applications of the waste glass are related to the manufacturing of different products like conventional ceramics, aggregates and cements [19,20]. Some of the technologies involve high temperature treatment of the CRT waste glass leading to ceramic or glass composites used in the construction industry, mainly to manufacture bricks and roof tiles [21,22]. The CRT waste glass cullet can be also used in the metallurgical industry to produce ferro-silicates in the form of slag [23]. Other methods have also been developed for the use of waste CRT glass in the production of floor coverings and chemical resistant compounds [24].

Unfortunately, the amount of waste material that can be recycled in the above mentioned technologies is limited due to the fact that waste CRT glass is used without preliminary separation of harmful components, involving higher environmental risks, operation and maintenance costs [25,26]. In order to overcome this drawback, associated with waste CRT glass recycling, there have been attempts to remove the hazardous components like lead employing different hydrometallurgical and pyrometallurgical processes [27,28,29]. These processes present some major disadvantages like insufficient lead removal efficiency and polluting byproducts, which can be more harmful than the treated waste material [30]. Moreover, many of the studies presented in the literature lack a comprehensive overview, by not assessing both the technical performance and environmental impact of the processes, which is necessary to draw global conclusions [29,31].

In view of the above discussion, the recovery of lead from waste CRT glasses was achieved by acetic acid leaching of Pb coupled with the simultaneous electrowinning of a high purity Pb deposit and regeneration of the leaching agent. The novel process concept defined and assessed in the current paper ensures high technical performance with a low environmental impact, based on the influence of acetic acid concentration, recirculation flow rate and current density on different key performance indicators and GEIs values.

## 2. Materials and Methods

### 2.1. Thermal Treatment of CRT Glass Samples

In a preliminary step the CRT glass samples with the composition presented in Table 1 were ground to a fine powder (3.5–5.5 μm) in order to promote the reaction between the CRT glasses samples and Na_2_CO_3_. All tests were carried out in an electrical furnace at 1000 °C with duration of 30 min, combining 10 g of CRT glass with 24 g of Na_2_CO_3_. After the thermal treatment the cooled samples were washed with 100 mL of distillated water in order to selectively remove the soluble silicates and hydroxides.

### 2.2. Lead Dissolution Process

The solid material obtained from the washing step was dissolved, over a period of one hour, in 150 mL of acetic acid (CH_3_COOH) solution using a chemical reactor equipped with a stirrer operated isothermally at 80 °C. In order to determine the optimal concentration of acetic acid for the dissolution process, the experiments were performed at different concentrations of CH_3_COOH in the range of 0.2–1 M. Glacial acetic acid of analytical purity and bidistilled water were used to obtain the leaching solutions. Finally, the solution was filtered in order to separate it from the precipitated solid residues which contained mainly SiO_2_. The lead concentration in the samples taken during the dissolution was determined using an atomic absorption spectrophotometer.

### 2.3. Electrochemical Process Description

The installation used for lead electroextraction consisted of a storage tank of the processed solution, connected in series with a divided electrochemical reactor (ER), operated in galvanostatic mode. Recirculation of the solution between the storage tank and ER was performed with a peristaltic pump. The cathode was made of stainless-steel plates and the anode was made of graphite. Two Ag/AgCl/KCl_sat_ reference electrodes were used to measure the cathodic and anodic potentials. All tests were performed at 22 °C for two hours using a 150 mL of electrolyte containing 9.25 g/L Pb^2+^, 3.91 g/L Mg(CH_3_COO)_2_ and 7.24 g/L Ca(CH_3_COO)_2_ which correspond to the final composition of the leaching solution. The experiments were performed at different flow rates (15, 30 and 45 mL/min) and current densities (4, 8 and 12 mA/cm^2^). Experiments also involved the use of a computer-controlled DC power supply, and LabVIEW software for process control and data acquisition. The obtained Pb deposit was dissolved in concentrated HNO_3_ to determine the amount and purity of lead deposited. The concentration of lead in the solutions at the end of the experiment was determined using an atomic absorption spectrophotometer.

### 2.4. Performance Indicators of the Lead Recovery Process

The performance of the dissolution and electro-extraction processes was evaluated on the basis of technical performance indicators:

- **Dissolution degree (%)** was defined as the ratio between the amount of dissolved lead and the initial amount of lead in the processed samples.
- **Efficiency of CH_3_COOH utilization (%)** is the ratio of the amount of CH_3_COOH consumed in the dissolution process and the initial amount of CH_3_COOH in the solution.
- **Specific acetic acid consumption (kg CH_3_COOH/kg Pb)** indicates the amount of CH_3_COOH consumed to dissolve one kilogram of Pb from the processed waste.
- **Extraction degree (%)** was calculated as the ratio of the quantity of electrodeposited lead and the initial amount of lead in the electrolyte.
- **Current efficiency (%)** was defined as the ratio of the amount of electricity used to form the cathode deposit and the total amount of electricity consumed in the process.
- **Specific energy consumption for the cathodic process (kWh/kg Pb)** indicates the amount of energy used to form one kilogram of Pb deposit.
- **Specific energy consumption for the anode process (kWh/kg CH_3_COOH)** indicates the amount of energy required to produce one kilogram of CH_3_COOH.

## 3. Results and Discussions

### 3.1. Dissolution of Lead from Pretreated CRT Glass

**Figure 1:**
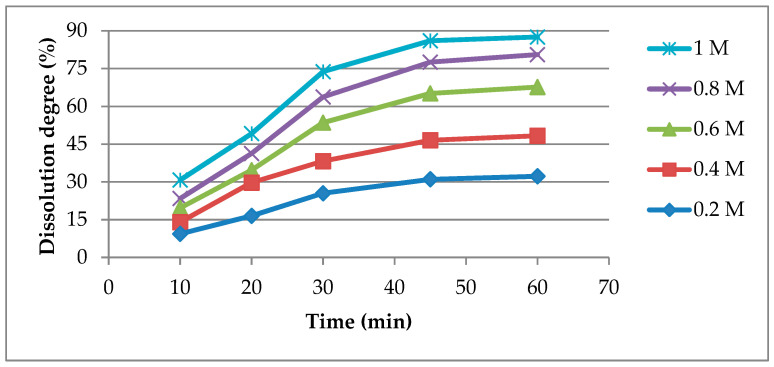
Dissolution degree vs. time at different CH_3_COOH concentrations.

In order to determine the optimal operating conditions for the dissolution process, the evolution of the dissolution degree at different acetic acid concentrations was quantified. It can be seen from Figure 1 that the dissolution degree increases over time at all CH_3_COOH concentrations, the final value being almost three times higher than the initial one. The results also show that the concentration of the leaching agent has a decisive influence on the dissolution rate, since at the concentration of 1 M CH_3_COOH the dissolution degree is three times higher than at 0.2 M CH_3_COOH. However, the dissolution degree values increase only with 30% between 0.6 and 1 M CH_3_COOH while between 0.2 and 0.6 M CH_3_COOH they increase 110%, which means that above 0.6 M CH_3_COOH there is no significant gain in efficiency.

**Figure 2:**
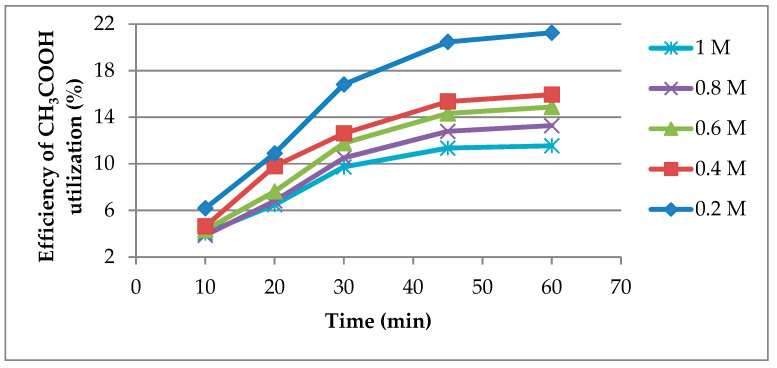
Efficiency of CH_3_COOH utilization vs. time at different CH_3_COOH concentrations.

In addition to increasing the dissolution degree with increasing CH_3_COOH concentration, it is important to increase the conversion of the leaching agent in order to exploit the full potential of the leaching solution. To highlight this aspect, the efficiency of CH_3_COOH utilization was determined, which indicates how much of the leaching agent was converted under different experimental conditions compared to what could theoretically be used for lead dissolution, taking into account the initial amount of CH_3_COOH in the solution. The results from Figure 2 show that the efficiency of CH_3_COOH utilization is diminished by increasing the concentration of CH_3_COOH, the maximum value being reached at a concentration of 0.2 M acetic acid.

**Figure 3:**
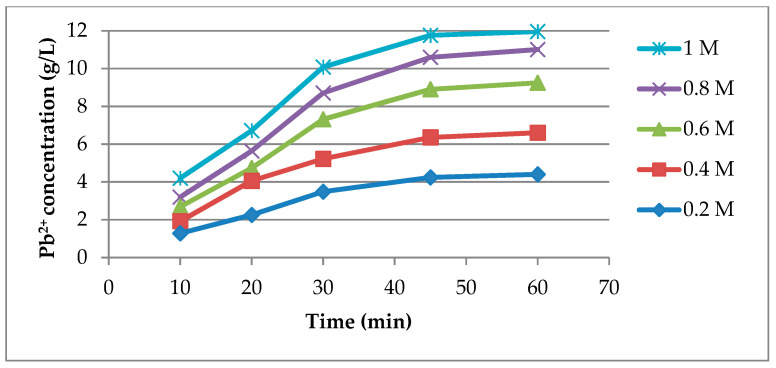
Pb^2+^ concentration profile at different CH_3_COOH concentrations.

This tendency can be attributed to a partial order of reaction regarding CH_3_COOH concentration which means that the amount of acetic acid transformed in the leaching reaction does not increases proportional with the increase in initial CH_3_COOH concentration. The concentration profiles of Pb^2+^ shown in Figure 3 sustain the above explanation, considering that the total amount of lead dissolved increases only 171% by increasing the initial concentration of CH_3_COOH by 400%.

**Figure 4:**
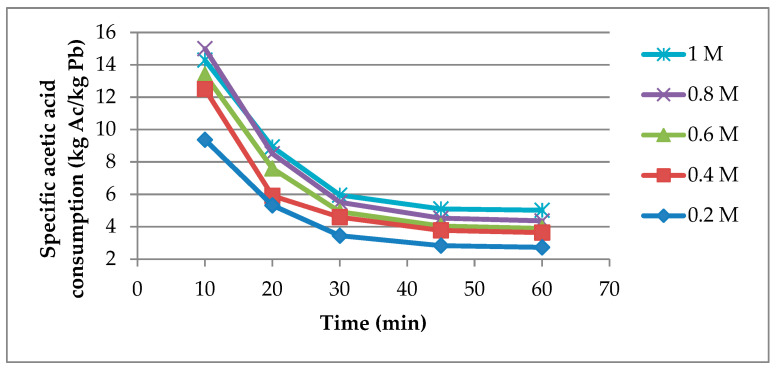
Specific acetic acid consumption vs. time at different CH_3_COOH concentrations.

Considering that the above evaluated performance indicators give contradictory conclusions regarding the optimal CH_3_COOH concentrations, the specific acetic acid consumption for the leaching process was determined which gives a comprehensive overview on the efficiency of the process. The specific acetic acid consumption values, Figure 4, show that the lowest concentration (0.2 M) of CH_3_COOH allows the most efficient use of the amount of leaching agent present in the system. In contrast, according to Figure 3, the final Pb^2+^ concentration is the lowest at 0.2 M of CH_3_COOH which would not ensure the most favorable conditions for the electrodeposition process of lead. Therefore, the intermediate concentration of 0.6 M CH_3_COOH would be a better option in comparison to 0.2 M CH_3_COOH, considering that the obtained final Pb^2+^ concentration (9.25 g/L) represents 80% of the maximum achievable concentration under these dissolution conditions.

Additionally, taking into account the ecological aspects, to ensure a relatively advanced removal of lead from CRT waste, but with a reasonable yield, the 0.6 M CH_3_COOH value can be considered as the optimal concentration for the dissolution process.

### 3.2. Lead Electrodeposition and CH_3_COOH Regeneration

The electrochemical recovery of lead from the leach solutions involved as main reactions the simultaneous deposition of Pb at the cathode and CH_3_COOH regeneration at the anode. Considering the position of Pb in the electrochemical series of metals, its formation at the cathode is accompanied by the hydrogen evolution reaction.

Cathode:

(1) $${\mathrm{Pb}}^{2+}+2e^{-}\to \mathrm{Pb}$$

(2) $$2H^{+}+2e^{-}\to H_{2}$$

Anode:

(3) $$2H_{2}O\to 4H^{+}+O_{2}+4e^{-}$$

Chemical reaction:

(4) $$H^{+}+{\mathrm{CH}}_{3}{\mathrm{COO}}^{-}⇄{\mathrm{CH}}_{3}\mathrm{COOH}$$

**Figure 5:**
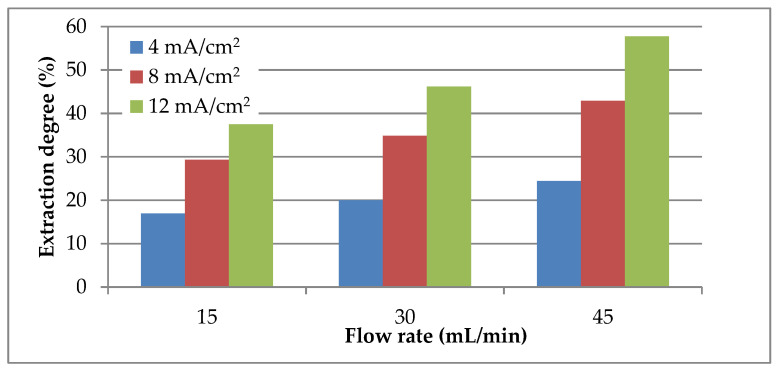
Influence of electrolyte flow rate and current density on lead extraction degree.

According to the results, Figure 5, the extraction degree values increase with the increase in electrolyte flow rate and current density reaching the maximum value at 45 mL/min and 12 mA/cm^2^. Additionally, the results reveal the fact that the extraction degree is more strongly dependent on current density than electrolyte flow rate. It can be observed, Figure 5, that the extraction degree values almost double with the increase in current density by three times while for the same increase in flow rate at constant current density increases the extraction degree by only 32%.

**Figure 6:**
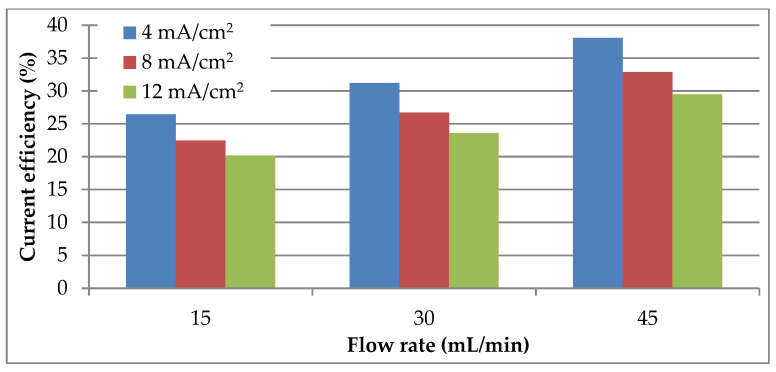
Evolution of current efficiency with electrolyte flow rate at different current densities.

As can be seen in Figure 6, in contrast to the evolution of extraction degree, the cathodic current efficiency decreases as the current density increases, while the increase in electrolyte flow rate has a positive impact. This can be explained by the fact that high current densities favor the hydrogen discharge reaction, which leads to a decrease in the cathodic current efficiency by 20–23% between the current densities of 4 and 12 mA/cm^2^. However, the experimental data show that the impact of the secondary cathodic reaction is even lower as the electrolyte flow rate is higher. In view of this tendency the maximum cathodic current efficiency (38.08%) was obtained at the highest flow rate (45 mL/min) and the lowest current density.

**Figure 7:**
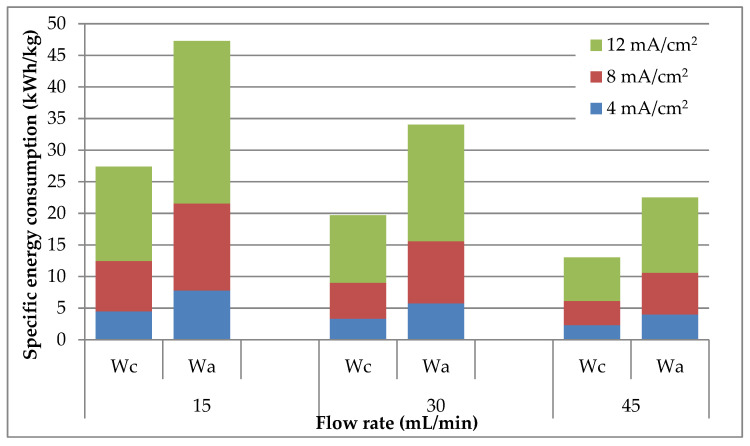
Specific energy consumption values for lead electrodeposition (Wc) and CH_3_COOH regeneration (Wa) at different electrolyte flow rates and current densities.

Since the most important performance criterion in the performance of an electrochemical process is the specific energy consumption, this parameter was evaluated for both lead deposition and regeneration of the leaching agent. From Figure 7 it can be seen that the specific energy consumption for the cathodic process depends more strongly on the current density than on the electrolyte flow rate and varies in the opposite direction with the two operating parameters. Increasing the current density by three times increases the specific energy consumption of the electrodeposition process by 197–231%, while the same variation of the electrolyte flow reduces it by about 100%. The beneficial impact of flow rate increase can be attributed to the more intensive transportation of Pb^2+^ ions to the cathode surface which reduces the corresponding mass transport potential.

Similar conclusions can be reached related to the influence of the operating parameters on the evolution of the specific energy consumption for acetic acid regeneration. In contrast, Figure 7 reveals that the specific energy consumption of the anodic process is almost twice as high as for the cathodic one. This is due to the lower molar mass of acetic acid than lead, which leads to the generation of a lower amount of CH_3_COOH by consuming the same amount of electricity.

The influence of the operating conditions on the performance of the lead electrodeposition process at the cathode and the CH_3_COOH regeneration at the anode is also quantified by the thermodynamic parameters, Table 2, of the electrochemical process. Electrode potentials confirm that lead deposition and oxygen discharge are the main electrochemical reactions, and their increase with increasing current density indicates the negative impact of the current density on the electrode over potentials. As can be seen from Table 2, increasing the current density also leads to higher ohmic drops, which leads to the increase in the cell voltage. In contrast, increasing flow rates reduce both electrode potentials and cell voltage, indifferent of the current density value, due to increased transport of electrochemically active species to the reaction surface.

Based on the above discussions, the optimal operating conditions were obtained at a flow rate of 45 mL/min and a current density of 4 mA/cm^2^, due to the fact that the specific energy consumption for both main electrochemical processes, Figure 7, attain the lowest values.

### 3.3. Environmental Assessment of the Lead Recovery Process

The environmental assessment was performed using the Biwer–Heinzle method [32,33] which is easily applicable in the early phases of process development and reveals the contribution of each input and output substance to the overall environmental impact of the lead recovery process.

**Figure 8:**
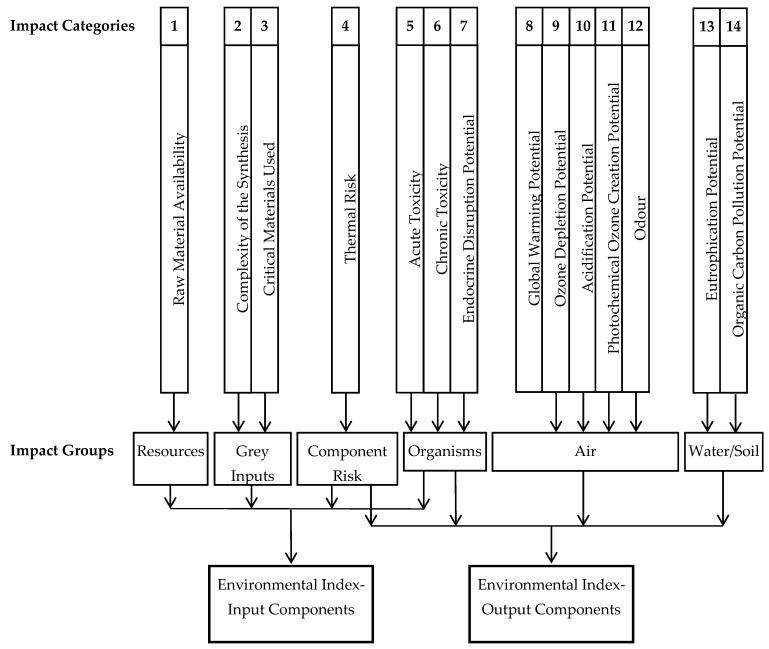
Schematic representation of the Biwer–Heinzle method.

In accordance with the Biwer–Heinzle method (Figure 8) the environmental factors were obtained from 6 impact groups which contained 14 impact categories. All of the components involved in the lead recovery process were allocated to a class *A*, *B* or *C* in each impact category (*A* = 1—highly toxic substances, *B* = 0.3—less toxic substances, *C* = 0—non-toxic substances) [32]. Next, the input and output environmental indices were determined by combining the obtained environmental factors with the mass indices resulting from the mass balance data corresponding with the processing of 10 kg/h CRT glass in the identified optimal conditions. Finally, the overall environmental impact of the lead recovery process was evaluated based on the General Effect Indices (GEIs) calculated by dividing the sum of environmental indices by the total mass indices [33].

Among the input materials (Table 3), water has the lowest environmental impact considering that it was allocated to class *C* in all seven impact categories. In contrast, CH_3_COOH was assigned to class *B* for its acute toxicity, thermal risk and raw material availability associated with their production. The processed waste CRT glass was assigned to class *B* in impact category 5 and 6 because Pb can cause serious health issues. For the same reason, in the case of the output streams, Pb was assigned to class *B* in impact category 5 and 6 together. An output stream with similar environmental impact is the waste acetic acid solution which was also assigned to class *B* in impact categories 4, 5, 11 and 14 regarding its impact on air, soil and water pollution. Considering the global warming potential of CO_2_, it was assigned to class *B* in impact category 8. The other output streams, H_2_O and SiO_2_ have the lowest environmental impact, considering that they were allocated to class *C* in all 11 impact categories, being valuable secondary products of the developed process together with the resulting lead acetate, calcium acetate and magnesium acetate.

The *GEI*s values from Table 4 indicate that the environmental impact of the output streams is lower than for the input streams, which means that the developed process lowers the environmental impact of waste CRT glass through the recovery of lead.

Since the input and output streams have *GEI*s values close to the minimum possible (0), according to the Biwer–Heinzle method, it means that globally the process has low environmental impact. Nevertheless, caution and special protective measures must be applied when handling concentrated CH_3_COOH solutions.

## 4. Conclusions

The results demonstrate that the developed combined chemical–electrochemical process can be efficiently applied for the recovery of Pb from waste CRT glass in the form of metallic Pb. It was found that the Pb dissolution process from the pre-treated waste CRT glass samples is most effective at a concentration of 0.6 M CH_3_COOH, a consideration which ensures equilibrium between the yield of dissolution process and leaching agent consumption. Based on the specific energy consumption, it can be concluded that the electrochemical process is carried out with the highest performance at a flow rate of 45 mL/min and a current density of 4 mA/cm^2^, leading to the formation of a high purity Pb deposit (99.98 wt.%) and CH_3_COOH regeneration. In the identified operating conditions, the amount of metallic lead recovered in one hour of processing represents ~10% of the lead present in the treated waste material the rest is in form of dissolved lead acetate.

The environmental impact assessment of the Pb recovery process was performed successfully in the early phases of process development by applying the Biwer–Heinzle method and the corresponding mass balance data for the treatment of 10 kg/h waste CRT glass. Based on the GEIs values obtained for the input and output streams, it can be stated as an overall conclusion that the novel approach for the recovery of lead from waste CRT glass proved to be a promising alternative with low environmental impact. Still, further studies are recommended in order to model, simulate and scale up the process for higher production and assess its economic performance.

## Figures and Tables

**Table 1:** Cathode-ray tube (CRT) glass composition.

Component	SiO_2_	TiO_2_	Fe_2_O_3_	Al_2_O_3_	CaO	MgO	Na_2_O	K_2_O	PbO	Other Trace Elements
**Concentration, wt.%**	55.6	0.2	0.2	2.96	3.85	1.65	6.25	6.75	22.08	0.46

**Table 2:** Thermodynamic parameters of the electrochemical process.

Flow Rate, mL/min	E_b_, V			ε_c_, V			ε_a_, V		
	i, mA/cm^2^			i, mA/cm^2^			i, mA/cm^2^		
	4	8	12	4	8	12	4	8	12
**15**	2.30	3.46	5.81	−0.2	−0.34	−0.74	1.62	1.96	1.98
**30**	2.01	2.94	4.87	−0.15	−0.21	−0.73	1.50	1.75	2.32
**45**	1.71	2.42	3.94	−0.07	−0.36	−0.66	1.43	1.67	2.47

E_b_—cell voltage; ε_c_—cathode potential; ε_a_—anode potential; i—current density.

**Table 3:** Input impact assessment.

Input			Impact Categories							Environmental Factors	Environmental Index
Streams	Quantity (kg/h)	Mass Index	1	2	3	4	5	6	7	*EF*	*EI*
Waste CRT	10	4.96	C	C	C	C	B	B	C	0.075	0.372
Sodium carbonate	7.8	3.87	C	C	C	B	C	C	C	0.075	0.290
Acetic acid	1.55	0.77	B	C	C	B	B	C	C	0.225	0.173
Water	50	24.81	B	C	C	B	B	B	C	0	0.000
Total:	**69.35**	**34.41**		Environmental Index, *EI_inputs_*:							**0.835**
				General Effect Index, *GEI_inputs_*:							**0.024**

**Table 4:** Output impact assessment.

Output			Impact Categories											Environmental Factors	Environmental Index
Streams	Quantity (kg/h)	Mass Index	4	5	6	7	8	9	10	11	12	13	14	*EF*	*EI*
Lead	2.02	**1.00**	C	B	B	C	C	C	C	C	C	C	C	0.075	0.075
Lead acetate	0.09	0.05	C	B	B	C	C	C	C	C	C	C	C	0.075	0.004
Calcium acetate	1.21	0.60	C	B	C	C	C	C	C	C	C	C	C	0.075	0.045
Magnesium acetate	0.62	0.31	C	B	C	C	C	C	C	C	C	C	C	0.075	0.023
Slicon dioxide	1.27	0.63	C	C	C	C	C	C	C	C	C	C	C	0	0.000
Waste acetic acid	1.16	0.57	B	B	C	C	C	C	C	B	C	C	B	0.3	0.172
CO_2_	3.24	1.61	C	C	C	C	B	C	C	C	C	C	C	0.075	0.121
Gases (O_2_, H_2_,...)	3.19	1.58	C	C	C	C	B	C	C	C	C	C	C	0.075	0.119
Wastewater	56.55	28.06	C	C	C	C	C	C	C	C	C	C	C	0	0.000
Total:	**69.35**	**34.41**					Environmental Index, *EI_outputs_*:								**0.558**
							General Effect Index, *GEI_ouputs_*:								**0.016**

## Data Availability

Data sharing is not applicable to this article.
